# Deep Learning Approaches on Defect Detection in High Resolution Aerial Images of Insulators

**DOI:** 10.3390/s21041033

**Published:** 2021-02-03

**Authors:** Qiaodi Wen, Ziqi Luo, Ruitao Chen, Yifan Yang, Guofa Li

**Affiliations:** Institute of Human Factors and Ergonomics, College of Mechatronics and Control Engineering, Shenzhen University, Shenzhen 518060, China; wenqiaodi2018@email.szu.edu.cn (Q.W.); 2018112059@email.szu.edu.cn (Z.L.); 2018112139@email.szu.edu.cn (R.C.); 1810294048@email.szu.edu.cn (Y.Y.)

**Keywords:** deep learning, defect detection, power inspection, insulator

## Abstract

By detecting the defect location in high-resolution insulator images collected by unmanned aerial vehicle (UAV) in various environments, the occurrence of power failure can be timely detected and the caused economic loss can be reduced. However, the accuracies of existing detection methods are greatly limited by the complex background interference and small target detection. To solve this problem, two deep learning methods based on Faster R-CNN (faster region-based convolutional neural network) are proposed in this paper, namely Exact R-CNN (exact region-based convolutional neural network) and CME-CNN (cascade the mask extraction and exact region-based convolutional neural network). Firstly, we proposed an Exact R-CNN based on a series of advanced techniques including FPN (feature pyramid network), cascade regression, and GIoU (generalized intersection over union). RoI Align (region of interest align) is introduced to replace RoI pooling (region of interest pooling) to address the misalignment problem, and the depthwise separable convolution and linear bottleneck are introduced to reduce the computational burden. Secondly, a new pipeline is innovatively proposed to improve the performance of insulator defect detection, namely CME-CNN. In our proposed CME-CNN, an insulator mask image is firstly generated to eliminate the complex background by using an encoder-decoder mask extraction network, and then the Exact R-CNN is used to detect the insulator defects. The experimental results show that our proposed method can effectively detect insulator defects, and its accuracy is better than the examined mainstream target detection algorithms.

## 1. Introduction

Electricity is one of the most essential elements to make the world go around, and the transmission of high-voltage electricity is very important for the practical use of it. In the transmission of high-voltage electricity, electrical insulators are used to support and separate electrical conductors without allowing current through themselves. Usually, insulators are exposed to the harsh environment of strong electric field with all the severe weather conditions such as blazing sunlight, typhoons or hurricanes, thunderstorms, freezing rain, and snowstorms. The harsh environment will make the insulators vulnerable damaged, which will threaten the safety of power grid systems and the use of electricity [1]. Therefore, it is necessary to develop effective methods for insulator defect detection to ensure the safe and reliable electric power transmission [2].

The current defect detection methods can be divided into three categories including physical methods [3,4], traditional vision-based methods [5,6,7,8,9,10,11], deep learning based methods [12,13,14].

The physical methods mainly include the ultrasonic wave method [3] and the ultraviolet pulse method [4] based on manual operation. In [3], the authors proposed an ultrasonic wave method for detection by receiving and processing ultrasonic signals between 20–40 kHz and 80–140 kHz because ultrasound at these frequencies mostly be emitted from the insulator failure location. In [4], the authors introduced an ultraviolet pulse method which identified the defect location by detecting the discharge of defective insulators. However, in practical application, the physical methods are easily affected by solar radiation, meteorological conditions, surrounding heat source, distance and other factors. In recent years, electric power inspection departments have begun to use UAV (unmanned aerial vehicle) instead of manual methods to detect insulator defect.

Among the vision-based insulator defect detection methods, the most frequently used ones are HOG + SVM [10] and Haar + AdaBoost [11]. As traditional detection algorithms, they mainly use sliding window to select the region of interest, extract the features of each window and then classify the feature samples to obtain the detection results. Besides, there are also methods based on contour features and gray similarity matching to classify intact and defective insulators [6]. For example, Zhang and Yang [2] proposed an insulators recognition method with curved smooth and weak textured (CSWT) surfaces. Li et al. [7] used the improved MPEG-7 EHD (edge histogram method) technique to recognize insulators. Wu et al. [5] used the global minimization active contour model (GMAC) for insulator segmentation. However, these methods are usually based accumulated experience to extract image features, so that they are inefficient, suffering from accuracy limitation and time-consuming problems.

With the continuous improvement of computer performance, detection methods based on deep learning frameworks have been widely used. It can effectively compensate the loss of feature information in the process of artificial image feature extraction and improve the efficiency of fault detection. A number of effective target detection algorithms have been proposed, such as Faster R-CNN (faster region-based convolutional neural network) [15], Yolo (you only look once) [16], SSD (single shot multibox detector) [17], DCNN (dynamic convolutional neural network) [18], etc. In reference [14], the authors proposed a method for insulator defect detection based on R-FCN (region-based fully convolutional network). This method is a two-stage object detection method based on RPN (regional recommendation network) and FCN [19] (fully convolutional network) with RPN generating the region proposals and FCN obtaining the feature maps. In reference [20], the proposed insulator defect detection method is based on the improved Faster R-CNN by using ResNet101 as the backbone. However, due to the fact that the high-resolution insulator images taken by UAV contain mostly irrelevant background information, the useful information of the insulator is not much in an image. In addition, the slender shape characteristics of insulators and different defect variations lead to the diverse and complex presentation of defects in images. Hence, the accuracy of the existing insulator detection methods still needs to be improved. The merits and demerits of the above-mentioned three method categories are summarized in Table 1.

Among these three categories, the physical methods are too labor work extensive, which makes it not practical for large-scale applications. The other two categories (i.e., traditional vision-based methods and deep learning based methods) mainly depend on camera systems. Most of the above-mentioned detection methods are based on monocular vision. They have a relatively simple hardware structure and are easily to be operated. However, these methods are disadvantaged by the instability of the available feature point extraction algorithms. Hence, extensive efforts based on multi vision had been conducted in displacement, crack, and deformation detection. In [21], the authors proposed a method for measuring the distance of a given target using deep learning and binocular vision methods, where target detection network methodology and geometric measurement theory were combined to obtain 3D (three-dimensional) target information. In [22], the authors presented a dynamic real-time method to detect surface deformation and full field strain in recycled aggregate concrete-filled steel tubular columns. In [23], the authors proposed a four-ocular vision system for 3D reconstruction of large-scale concrete-filled steel tube under complex testing conditions. Compared with the methods based on monocular vision, these technologies based on multi vision consistently demonstrate high accuracy, stability, and feasibility. However, the multi vision methods are usually based on more expensive equipment than monocular vision methods, and the weight of multi vision systems are usually heavier than monocular vision systems. These disadvantages make the multi vision methods not as feasible as monocular vision methods to be applied in UAV. Therefore, the methodology developed in this study is based on monocular vision.

To alleviate the low accuracy problems of the above-reviewed deep learning-based methods, two methods inspired by Faster R-CNN for insulator defect detection are proposed. A series of advanced techniques are used to improve the network performance including RoI Align (region of interest align), cascade regression, and FPN (feature pyramid network). Besides, a new detection pipeline is proposed to improve the detection accuracy, following the idea that reducing the inference of background information could improve the detection performance. The main contributions of this study are listed as follows.

1.  A new detection network is proposed for the detection of insulator defects, and the method is verified to be effective on a self-collected dataset that has been released to be publicly available online. Our proposed method addresses the misalignment and huge computational burden problems in Faster R-CNN, and advanced techniques including RoI Align, cascade regression and FPN are used to improve the network performance.
2.  A new two-stage defect detection pipeline is proposed. In the first stage, the insulator mask image is generated to reduce the interference of the background information in images by the encoder-decoder mask extraction network. The Exact R-CNN (exact region-based convolutional neural network) is then used to detect insulator defects in the second stage. Compared with the traditional target detection method, this two-stage pipeline can greatly eliminate the interference of background features on the target detection, and greatly improve the accuracy of target detection.

## 2. Related Work

The deep learning-based methods developed for object detection can be mainly divided into two categories: the one-stage method [16,17,24] and the two-stage method [15,20,25]. The one-stage method directly classifies the object and renders a regression of the object location from the raw image. The two-stage method divides the detection problem into two steps. The region of interest where the object may be located in is firstly extracted from the image, and then the candidate regions are corrected and identified. The one-stage method runs faster, but it has a relatively lower accuracy compared with the two-stage method. The methods developed in this study are based on a typical two-state method, namely Faster R-CNN. The following knowledge from the previous literature is used for the development of our proposed approaches.

### 2.1. Faster R-CNN

**Figure 1:**
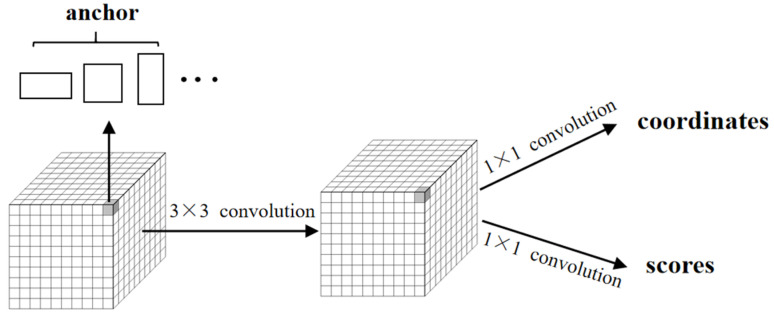
The schematic framework of RPN (regional recommendation network).

Faster R-CNN is an improvement of Fast R-CNN (fast region-based convolutional neural network) [26] and uses RPN instead of selective searching method to generate candidate regions. In the process of candidate regions generation, multiple bounding boxes are generated for each pixel in the feature map according to the offset of the corresponding anchors in different scales. When the candidate regions are selected, the feature maps are sent into two networks for classification and regression respectively. The classification in RPN is used to distinguish whether the corresponding anchor belongs to the foreground or the background, and the regression in RPN is to predict the offset coordinates between the anchor box and the ground truth. The schematic framework of RPN is shown in Figure 1.

**Figure 2:**
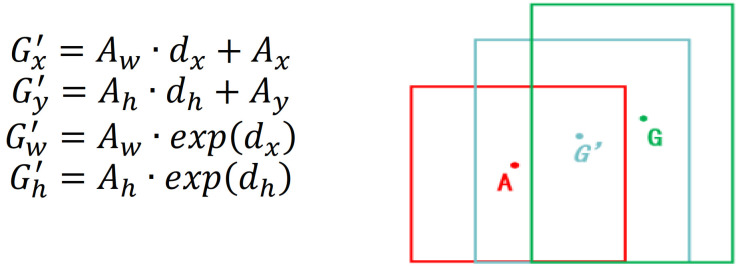
Process of correcting the anchor box.

A box can be described by a four-dimensional vector $\left(x,y,w,h\right)$ where x and y are the coordinates of the center point in the box and w and h are the width and height of the box, respectively. Set the given anchor box as $A=\left(A_{x},A_{y},A_{w},A_{h}\right)$ and the ground truth as $G=\left(G_{x},G_{y},G_{w},G_{y}\right)$. By learning the offsets of the dimensional vector, the predicted box $G^{\prime }$ obtained from A after translation and scaling is closer to G. This process is shown in Figure 2.

**Figure 3:**
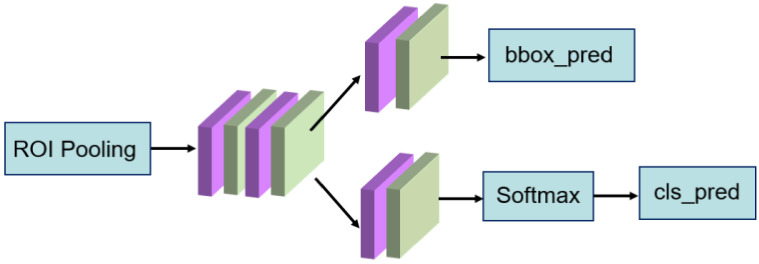
The framework of the re-regression and re-classification network.

After obtaining the candidate regions, the positive and negative samples are set by judging the threshold of intersection over union (IoU). If the IoU is higher than a given threshold, the anchor box is determined as the foreground, otherwise it belongs to the background. These candidate regions generated by RPN, namely RoI (region of interest), are further mapped into the feature maps which are then sent into the detection network. Due to the different size and length of the feature map, uniform size could be achieved through RoI pooling (region of interest pooling) to facilitate the subsequent re-classification and re-regression. The framework of the re-classification and re-regression network is shown in Figure 3.

The loss function of the Faster R-CNN is defined as shown in Formulas (1)–(5).

(1) $$L\left\{p_{i}\right\},\left\{t_{i}\right\}=\frac{1}{L_{cls}\left(p_{i},p_{i}^{*}\right)}+\lambda \frac{1}{N_{reg}}\underset{i}{\sum }p^{*}L_{reg}\left(t_{i},t_{i}^{*}\right)$$

(2) $$L_{reg}\left(t_{i},t_{i}^{*}\right)=\underset{i\in \left\{x,y,w,h\right\}}{\sum }smooth_{L1}\left(t_{i},t_{i}^{*}\right)$$

(3) $$L_{cls}\left(p_{i},p_{i}^{*}\right)=-\overset{N}{\underset{i}{\sum }}\left(p_{i}\log p_{i}^{*}\right)+\left(1-p_{i}\right)\log\left(1-p_{i}^{*}\right)$$

(4) $$smooth_{L1}\left(x\right)=\left\{\begin{array}{c} 0.5x^{2}\;\;\;\;\;if\left|x\right|<1\\ \left|x\right|-0.5\;\;\;\;\;\;otherwise \end{array}\right.$$

(5) $$p_{i}=softmax\left(x\right)=\frac{e^{x_{i}}}{\sum_{j}e^{x_{i}}}$$

where $L\left(\left\{p_{i}\right\},\left\{t_{i}\right\}\right)$ is the loss function of Faster R-CNN, $L_{reg}\left(t_{i},t_{i}^{*}\right)$ is the loss function of regression, $L_{cls}\left(p_{i},p_{i}^{*}\right)$ is the logarithmic loss of both categories (goals and non-goals), $p_{i}$ is the probability of the anchor box belonging to foreground, $p_{i}^{*}$ is the ground truth label of the anchor box, $t_{i}$ is the predicted location coordinate of the anchor box, $t_{i}^{*}\;$ is the ground truth location coordinate of the anchor box, $N_{cls}$ is the size of mini-batch, $N_{reg}$ is the number of anchor boxes. In practical applications, there is always a big gap between $N_{cls}$ and $N_{reg}$, and the gap would always cause the loss function to be difficult to converge. Therefore, the parameter λ is introduced to balance these two elements. For more details of Faster R-CNN, please refer to [15].

### 2.2. Cascade R-CNN

**Figure 4:**
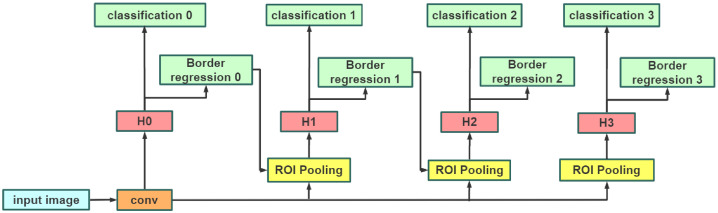
Framework of cascade R-CNN.

The detection models (e.g., SSD, Faster R-CNN) usually suffer the mismatch problem caused by the fixed IoU threshold (e.g., 0.5) to distinguish the positive and negative samples. Normally, a lower IoU threshold would generate more mismatch samples, while in contrast, a higher IoU threshold generates less. Therefore, in order to solve this mismatch problem, Cai et al. [27] proposed a multi-stage framework, namely cascade R-CNN. The network framework has multiple heads, and the threshold value of each network increases step by step. This network framework makes the output of each stage adapt to the threshold value of the next stage well, and it can reduce the probability of false positive samples and improve the effect of target detection. The framework of cascade R-CNN is shown in Figure 4.

### 2.3. Depthwise Separable Convolution

**Figure 5:**
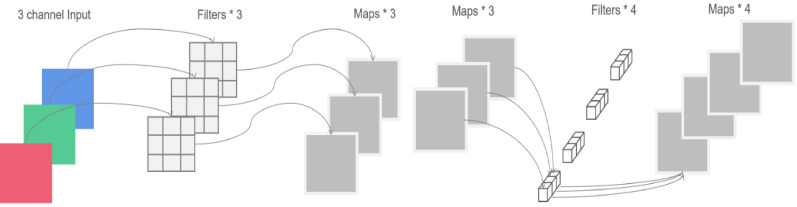
Framework of Depthwise Separable Convolution.

Depthwise separable convolution is a combination of depthwise and pointwise convolution. Its framework is shown in Figure 5. The depthwise convolution applies a filter to each input channel separately, while the pointwise convolution applies a 1 × 1 convolution to consolidate the output of depthwise convolution. In general, the computational cost of depthwise separable convolution is 8 to 9 times less than the standard convolution with decreased accuracy [28]. To alleviate this problem, a new architecture based on deeply separable convolution, namely linear bottlenecks and inverted residuals, was proposed.

### 2.4. Linear Bottleneck and Inverted Residuals

Chollet et al. [29] found that the performance of depthwise convolution after nonlinear activation function was worse than that after linear activation function. In another study [30], the researchers proved that in the low dimensional manifold of interest, the output would lose information through Relu function. The lower the dimension of the manifold of interest, the more information loss. To alleviate the problem mentioned above, linear bottleneck and inverted residual were proposed [30].

**Figure 6:**
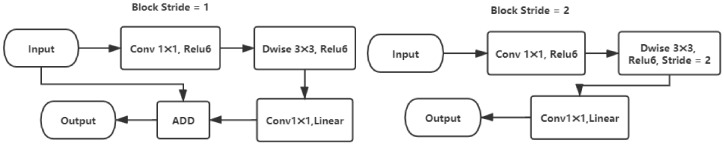
Linear bottlenecks and inverted residuals.

Its framework is shown in Figure 6, and it is implemented as follows: Conv1 × 1 (expansion) → Dwise3 × 3 (reduced computing costs) → Conv1 × 1 (projection). The purpose of the expansion layer is to reduce the lack of information because the use of nonlinear functions is inevitable. Linear activation function is used to minimize the information loss after dimension projection. Shortcuts are used directly between bottlenecks, which is different from ResNet [31].

### 2.5. U-Net

**Figure 7:**
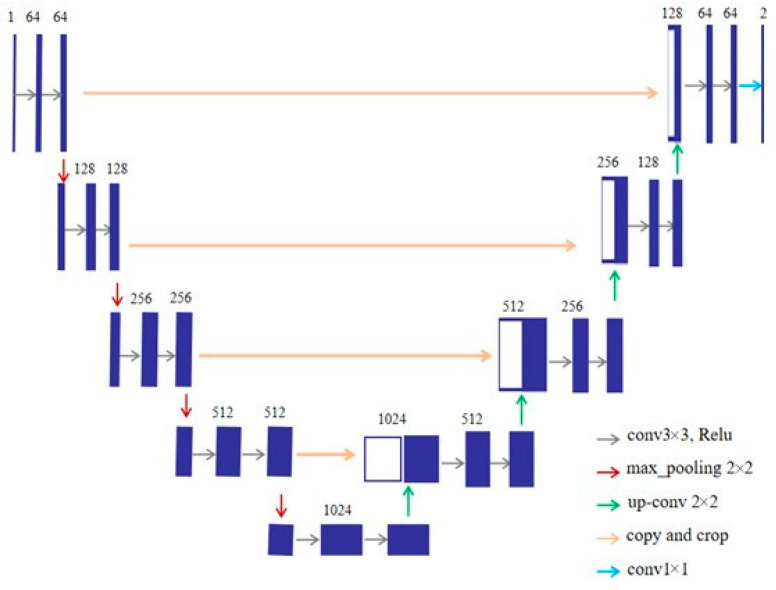
Framework of U-Net.

U-Net [32] is improved based on FCN. The encoder-decoder network framework of U-Net is shown in Figure 7. The encoder uses 3 × 3 convolution and pooling down sampling to extract image features and to reduce the spatial dimension. The decoder uses 3 × 3 convolution and pooling up sampling to repair the details and spatial dimensions of the image. In addition, the U-Net can combine the high-level features with the low-level features, so that more accurate segmentation results can be obtained.

## 3. Proposed Approaches

In this section, two models based on deep learning are proposed for insulator defect detection in high-resolution aerial images. The first model, namely Exact R-CNN, is inspired by the Faster R-CNN [15], but the network framework, the setting of super parameters, and the loss function are different from the Faster R-CNN. For simplicity, ERCN is used to represent Exact R-CNN. The second model, namely CME-CNN (cascade the mask extraction and exact region-based convolutional neural network), detects the defect of the insulator on the mask image of the information concentration through cascading an encoder-decoder mask extraction network and ERCN to improve the accuracy of the network. The depthwise separable convolution and linear bottlenecks are also used in CME-CNN to reduce the computational cost. Table 2 shows the comparison between the proposed methods and the traditional R-CNN methods. The source codes of our proposed approaches are available online [33].

### 3.1. Exact R-CNN

**Figure 8:**
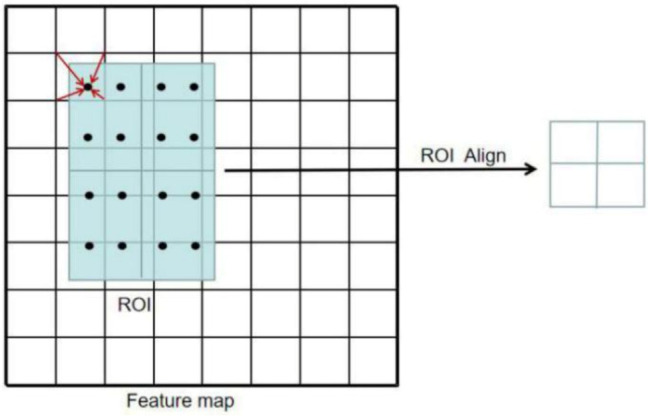
How RoI (region of interest) Align works.

**Figure 9:**
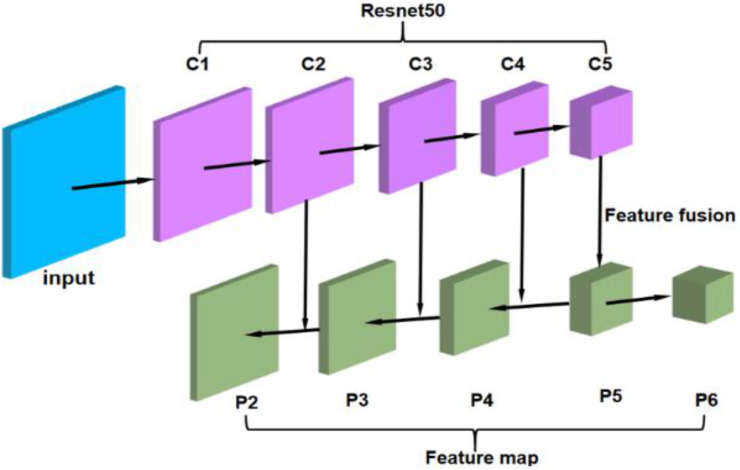
Framework of the improved backbone.

In the original article of Faster R-CNN, the backbone was inspired by Vgg16 [34]. Differently, Resnet50 is used in ERCN to replace the Vgg16. Compared with Vgg16, the deep framework of ResNet can not only extract more features, but also avoid the problem of gradient disappearing. This helps to promote convergence and model training. Considering that the features of insulator defect (small target) are easy to be ignored, the FPN framework [35] is adopted to extract both the semantic information of high-level features and the location information of the low-level features. Inspired by Mask R-CNN [36], the original RoI pooling can be changed to RoI Align to solve the misalignment problem caused by the twice quantization in the pooling operation and to improve the detection accuracy of small objects [37]. Figure 8 shows how RoI Align works. It uses bilinear interpolation to get the eigenvalues of floating-point coordinates. The improved backbone used in our method is shown in Figure 9, where C1~C5 are backbone ResNet50, and P1~P6 are the levels of feature maps after feature fusion.

**Figure 10:**
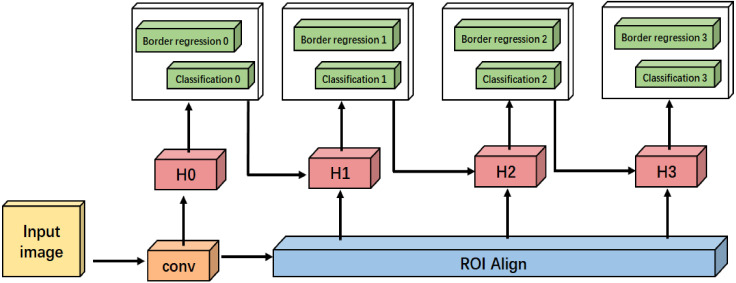
The improved framework of cascade R-CNN.

To solve the mismatch problem, cascade R-CNN is used to increase the IoU threshold level step by step, so that the proposals of each stage can better adapt to the high threshold of the next level, reducing the probability of false positive samples and improving the accuracy of target detection. Its improved framework is shown in Figure 10. Compared with Figure 4, the improved framework in Figure 10 replaces the RoI pooling with RoI Align.

The original Faster R-CNN used 9 types of anchor boxes with ratios of 0.5, 1, 2, and sizes of 128, 256, 512. However, due to the different datasets used, these ratios and sizes of anchor boxes are not suitable for slender insulator defects, the K-means++ [38] algorithm is used to cluster the bounding boxes of the insulator defects in our dataset to generate 9 groups of anchor boxes of different ratios and sizes that are more suitable for the characteristics of the insulator defects. Then the network performs fine-tuning on the basis of these anchor boxes to generate more accurate bounding boxes. When distinguishing between positive samples and negative samples, the IoU filter is changed to GIoU [39] filter because GIoU takes into account that the two bounding boxes do not overlap. This can reflect the overlap mode, so that the extracted area is more consistent with the target area.

**Figure 11:**
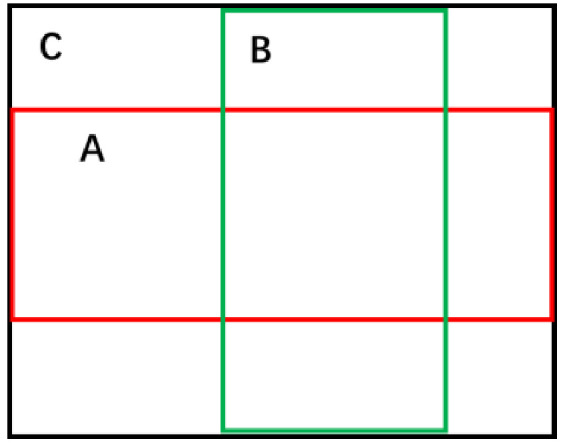
The calculation of GIoU.

The GIoU diagram is shown in Figure 11. For the red box A and the green box B, GIoU is calculated as defined in Formulas (6) and (7).

(6) $$IoU=\frac{\left|A\cap B\right|}{\left|A\cup B\right|}$$

(7) $$GIoU=IoU-\frac{\left|C-\left(A\cup B\right)\right|}{\left|C\right|}$$

The Smooth L1 is used as the loss function for the bounding box regression. It should be noted that the offset between the prediction and the target is regressed, hence the network output needs to be decoded to get the final frame coordinates. The decoding process is shown in Algorithm 1. In terms of the classification loss, we use the cross entropy function of the binary classes (i.e., insulator and background).

Considering that it is difficult to identify insulator defects on sample images with complex background environment, a cascaded network based on U-Net for improvement is proposed.

### 3.2. CME-CNN

**Figure 12:**
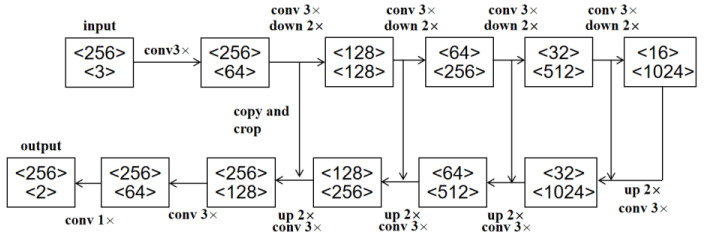
Selection and arrangement of successive convolutional layers in U-Net. The first number in each box represents the resolution of the feature map, and the second number represents the number of feature map channels.

In order to achieve more accurate detection of insulator defects, CME-CNN is to segment the insulators in the images to obtain the detailed information of the insulators without background information, and then use ERCN to detect the defect position. U-Net is used as the encoder-decoder mask extraction network to segment the insulators. The selection and arrangement of successive convolutional layers in U-Net are shown in Figure 12, which is consistent with [32]. Specifically, the encoder uses a 3 × 3 convolution and performs four down samplings. The decoder uses a 3 × 3 convolution and performs four up-sampling, splicing with low-level features in the up-sampling process. In this study, we use the method of padding zeros to make the size of feature map unchanged before and after convolution. Finally, a two-channel output including the background and target classification results is obtained. However, the complicated background of the insulator images and the various shapes and colors of the insulators make the segmentation task difficult. In order to further improve the encoder-decoder mask extraction network (U-Net) performance, the following improvements are proposed.

**Figure 13:**
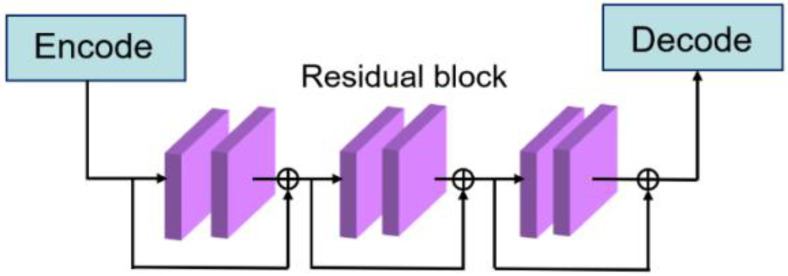
The residual blocks between the encoder and decoder.

In general, deepening the network can make it extract more features and enhance the performance of the network. However, if the network is too deep, it will bring a series of problems such as over fitting, gradient disappearing, and gradient explosion. Therefore, as shown in Figure 13, three residual blocks are added between the encoding and decoding process of the U-Net. Because the deep residual network is easier to optimize than the ordinary deep neural network, it can effectively avoid the occurrence of gradient disappearance or gradient explosion caused by the network layers being too deep [31]. By introducing residual blocks, a deeper network can be trained.
**Algorithm 1** Decode the output of the detection model**Input:**The output of dectection model,$Tensor=\left\{t_{1},\ldots ,t_{200}\right\},t_{i}\in R^{6};$**Output:**   $All\;bounding\;boxes\;B=\left\{b_{1},\ldots ,b_{200}\right\},b_{i}\in R^{4};$
   $And\;the\;corresponding\;score\;S=\left\{s_{i},\ldots ,s_{200}\right\}$, $s_{i}\in R^{1};$1:  **function Decode output**$\left(Tensor\right)$2:  **for**
$i\in \left[0,200\right]$
**do**3:    $x_{a},y_{a},h_{a},w_{a}\;are\;priori\;boxes\;attr$*/4:    $\left(x_{t},y_{t},w_{t},h_{t},bscore,pscore\right)\leftarrow t_{i};$5:    $xc_{p}\;=x_{t}\times w_{a}\left[i\right]+x_{a}\left[i\right];$6:    $yc_{p}=y_{t}\times \left[i\right]+x_{a}\left[i\right];$7:    $h_{p}=h_{a}\left[i\right]\times e^{ht};$8:    $w_{p}=w_{a}\left[i\right]\times e^{wt};$9:    $x_{min}=xc_{p}-w_{p}/2;\;$10:    $x_{max}=xc_{p}+w_{p}/2;$11:    $y_{min}=yc_{p}-h_{p}/2;$12:    $y_{max}=yc_{p}+h_{p}/2;$13:    $b_{i}=\left(x_{min},y_{min},x_{max},y_{max}\right)$:14:    $s_{i}=pscore$15:    **end for**16:  **return**
B,S17:  **end function**

**Figure 14:**
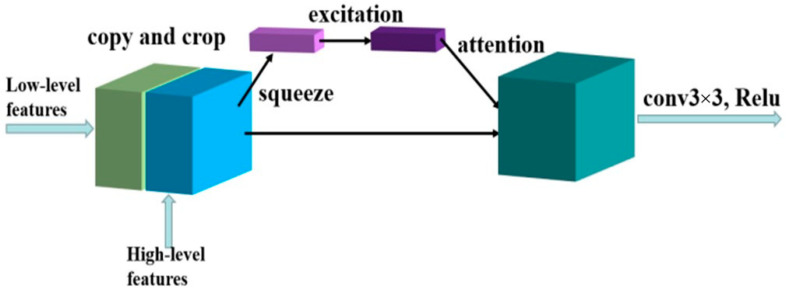
The framework of the squeeze and excitation block.

Although the high-level network contains more semantic information [35], the receptive field is larger, and the loss of image details is more severe because of the extensive pooling operations. Differently, the low-level network contains more edge, texture and other details, but it lacks semantic information and cannot classify accurately. The U-Net method simply spliced high-level features and low-level features together and cannot give different attention to low-level features and high-level features [32], which was not conducive to the accurate classification of pixels by neural networks. These problems will cause inaccurate detection, indicating that it needed to pay different attention to these characteristics. Therefore, the squeeze and excitation (SE) block is used to assign different weights to each channel when the underlying features and advanced features are stitched together [40,41]. The squeeze and excitation block is divided into three parts including squeeze, explanation, and attention. Please refer to Figure 14 for the detailed framework of this block.

The squeeze part means that all the eigenvalues in each channel are summed up and averaged, namely global average pooling, which is defined in Formula (8) as,

(8) $$z_{c}\;=\;F_{sq}\left(u_{c}\right)\frac{1}{H*W}\overset{H}{\underset{i=1}{\sum }}\overset{W}{\underset{j=1}{\sum }}u_{c}\left(i,j\right)$$

where $u_{c}$ is a channel of the feature map, H is the height of the feature map, and W is the width of the feature map.

As defined in Formula (9), the excitation part is to generate the attention weights from zero to one between channels.

(9) $$s=F_{ex}\left(z,W\right)=\sigma \left(g\left(z,W\right)\right)=\sigma \left(W_{2}\delta \left(W_{1}z\right)\right)$$

where δ means the Relu activation function, σ is the Sigmoid activation function, W1 and W2 are the weights that need to be learned. By learning these two weights, the one-dimensional activation weights can be obtained, and different channels of the feature maps are multiplied by the weights to enhance the focus on key channels.

Since the area occupied by an insulator is much less than the background, the imbalance of positive and negative samples during training would make the network difficult to learn the effective features of insulator defects. Therefore, the loss function of U-Net adopts focal loss [42] to reduce the weight of a large number of negative samples while training. The focal loss function is defined as,

(10) $$L_{fl}=\left\{\begin{array}{c} -a{\left(1-\gamma^{\prime }\right)}^{\gamma }\log\gamma^{\prime },\;\;\;\;\;\;\;\;\;\;\;\;\;\;\;\;\;\;\;\;\;\gamma =1\\ -\left(1-a\right){\gamma^{\prime }}^{\gamma }\log\left(1-\gamma^{\prime }\right),\;\;\;\;\;\;\;\;\;\gamma =0 \end{array}\right.$$

where α is used to balance the uneven proportion of positive and negative samples, γ adjusts the rate of weight reduction of simple samples. In reference [42], it performs well in target detection tasks when α = 0.25 and γ = 2. Following its parameter setting, α =0.25 and γ = 2 are set in the experiment.

**Figure 15:**
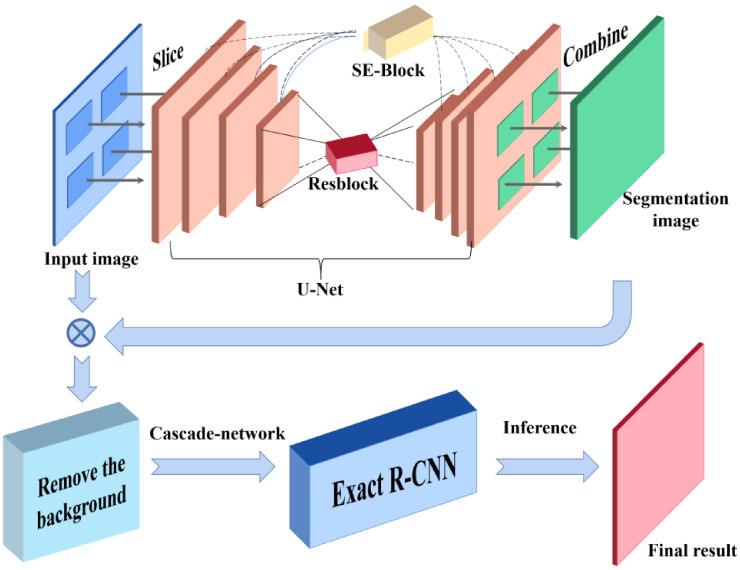
The general flowchart of our proposed CME-CNN (cascade the mask extraction and exact region-based convolutional neural network).

To decrease the computational cost caused by the cascading networks, depthwise separable convolution, linear bottlenecks and inverted residuals are adopted to reduce the number of model parameters while maintaining the accuracy [43]. By implementing all the above-introduced improvements and techniques, the general flowchart of our proposed detection framework is shown in Figure 15, and the corresponding pseudocodes are presented in Algorithms 2 and 3.
**Algorithm 2** The pseudocode of our proposed CME-CNN**Input:**   Dection model index u and e;$NMS\;threshold\;N_{t},\;Score\;threshold\;s;\;$**Output:**   The detection result imgBuffer; 
1: /*Bulid model and load model parameter*/2: $segamentation\;Model\;=\;nets\;Factory.getModel\left(u\right);\;$3. $detection\;Model\;=\;nets\;Factory.getModel\left(e\right);$4: $imgBuffer\;\leftarrow \left\{\;\right\};$5: /*Detection Framework Start*/6: **while**
detection Model On 
**do**7:    /* Get Img Frame*/8:    img=camera.getImgFrame();9:    /*Preprocess high resolution image*/10:    $imgBlocks=Preprocess\left(img\right)\;\;\;\;\;\;\;\;imgBlock\in R^{4\times 256\times 256\times 3}$11.    /*Morphological filter processing*/12:    $refineBlocks\;=\;Filter\left(maskBlocks\right),\;refineBlocks\in R^{4\times 256\times 256\times 3}$13:    /*Get the img without background */14:    $maskImg\;=\;Combine\left(refineBlocks.*imgBlocks\right),\;maskImg\in R^{1024\times 1024\times 3}$15.    $output=detection\;Model.detect\left(maskImg\right),$        $output\in R^{200\times 6}$16.    /* Decode the output*/17.    $Boxes,Score=Decode\;\;output\left(output\right)$18.    /*Non Maximum Suppression*/19.    $Boxes,Score=NMS\left(Boxes,Score,N_{t}\right)$20.    **for**
$each\;\left(box,score\right)\in \left(Boxes,Score\right)$
**do**21.        /*Draw the Bounding Boxes in*/22:        **if**
score≥s
**then**23:         $resultImg=Rectangle\left(reshapeImg,box\right)$24:        /*Append the result in img buffer*/25:           Imgbuffer←imgBuffer∪resultImg26:        **end if**27:    **end for**28:    **if**
detection Mode off
**then**29:        **break;**30:    **end if**31: **end while****Algorithm 3** Non-Maximum Suppression**Input:**    **Bounding boxes**$B=\{b_{1},\ldots ,b_{200}\}\;$, $\;b_{i}\in R^{4}$; $N_{t}$    $Scores=\left\{s_{1},\ldots ,s_{200}\right\}$, $s_{i}\in R^{1};$**Output:**    Bounding boxes D,score S;1: **function**
$NMS\left(B,S,N_{t}\right)$2: D←{ }3: **while**
 B # empty 
**do**4:    $m\leftarrow argmax\left(S\right)$5:    $M\leftarrow b_{m}$:6:    D←D∪M:7:    B←B−M:8:    **for**
$b_{i}\;in\;B\;$
**do**9:        **if IoU**$\left(M,b_{i}\right)>N_{t}$
**then**10:            $B\leftarrow B-b_{i}$:11:            $S\leftarrow S-S_{i}$:12:        **end if**13:    **end for**14: **end while**15: **return**
D,S16: **end function**

**Figure 16:**
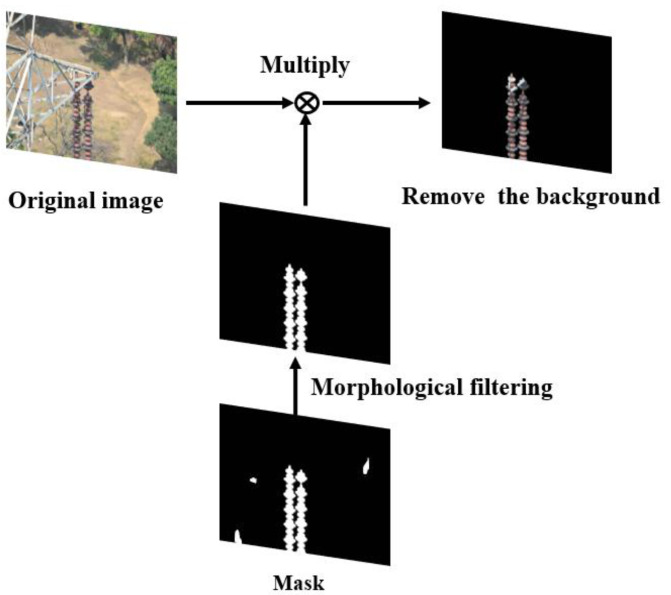
The mask schematic diagram.

As illustrated in Figure 15, high-resolution images are firstly cut into several low-resolution images, and then semantic segmentation algorithms (e.g., U-Net) are used to segment the low-resolution images. Next, the segmentation results are merged to get the insulator image after removing the background. Finally, target detection algorithms (e.g., ERCN) are used to detect the defect of insulators. The detailed process to remove the background is shown in Figure 16. As illustrated in the extracted mask in Figure 16, a lot of noise can be observed, thus the morphological filtering is used to solve this problem. Firstly, an open operation is conducted to remove small black dots in the white area, following by a close operation to remove small white dots in the black area. Then, the connected domain analysis is carried out to remove the connected domains with too small area to obtain a more accurate insulator mask image. Finally, the insulator image after background removal is obtained by multiplying the extracted mask image with the original image.

## 4. Experiment

**Figure 17:**
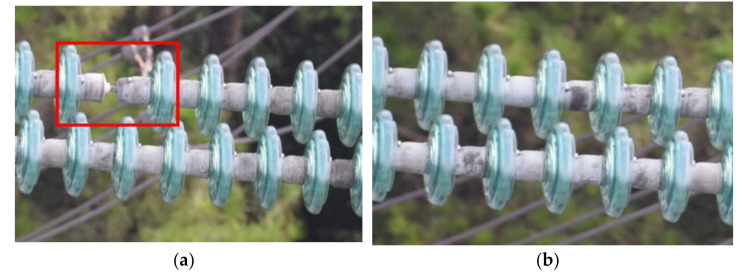
Examples of health insulators and insulators with defects. (**a**) Insulator with defects. (**b**) Health insulator.

To examine the effectiveness of our proposed methods, 879 high-resolution insulator images taken by UAV were collected from the Internet, of which 660 were used as the training set and the other 219 were used as the test set. The images in the data set were high-resolution images, with a resolution ranging from 3936 × 2624 to 7360 × 4912. Figure 17 shows two examples of health insulators and insulators with defects. All the images were processed on intel-i7 6700 K (4.0 GHz) with GTX 1080 and were compressed to 1024 × 1024. However, if the image size was adjusted directly, the aspect ratio of the original image would be damaged, resulting in the loss of texture, edge and other information in the image. To avoid this problem, the image was filled with black edges of length $\left(L-S\right)/2\;$(long side *L*, short side *S*) and used the bilinear interpolation method to reduce the images to 1024 × 1024. In this case, the aspect ratio of the original image could remain unchanged during the scaling process. For the encoder-decoder mask extraction network, four 256 × 256 image blocks containing all the image information are extracted from the 1024 × 1024 sample images.

**Figure 18:**
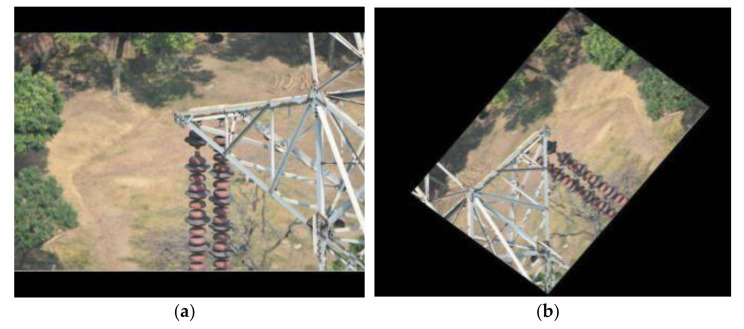

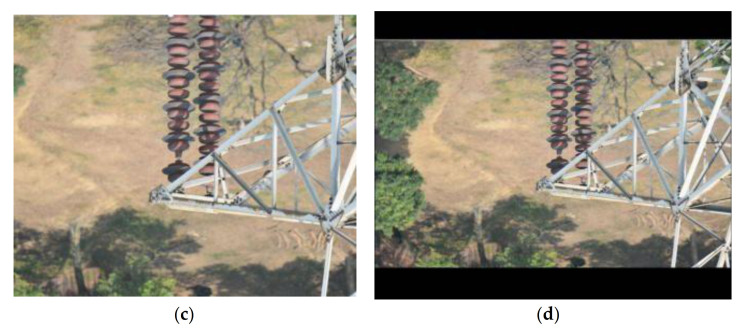
Examples of augmented images. (**a**) flipping. (**b**) random rotation. (**c**) random scaling. (**d**) reverse.

However, the number of images in our dataset was obviously not enough for the training of a reliable deep learning-based model. Therefore, image augmentation technologies were used to expand the dataset, including flipping, random rotation, random scaling and reverse. Figure 18 shows an example of these extended images. Finally, 6600 training set images and 2,190 test set images were obtained. In fact, our dataset could get enough images after augmentation, which largely enhanced the generalization ability of our models. The augmented dataset used in this study is publicly available online [33]. The following strategies were also used for network training:

(1)  Warmup strategy: In the beginning of training, the learning rate was not set as the initial value directly but increased it from a small value to the setting value by a constant amount at each iteration. This ramp avoids a sudden increase of the learning rate, allowing healthy convergence at the start of training. After the warmup, we went back to the original learning rate schedule [44].
(2)  Hard negative mining strategy: In the training of RPN, the negative samples that were easy to be classified (GioU < 0.3) were not included in the loss because they could not contribute much to the loss. Only the positive samples and negative samples with 0.3 < GioU < 0.5 that were difficult to be distinguished were included.
(3)  Sample balance strategy: The number of collected negative samples was far more than the number of positive samples. Hensman et al. [45] found that the distribution of the training data has a big impact on CNN performance and a balanced training set is optimal. Hence, the proportion of positive samples and negative samples for training was controlled to 1:1 to achieve better training performance.

The criteria defined in the PASCAL VOC 2012 competition was used to evaluate the predictions. When a prediction frames multiple insulator defects, the prediction will be assigned to the ground truth with the largest IoU. When multiple predictions frame the same insulator defect, they will be sorted according to the obtained confidences. All the predictions are compared with the ground truth for IoU calculation, and they will be considered as “match” when all IoU  ≥  0.5. Actually, to avoid the same insulator defect being detected by multiple predictions, only the result with the highest confidence was considered as the true positive.

When comparing the performance of different algorithms on our test set, the comparison indexes including average precision (AP), precision (P), recall rate (R) and frames per second (FPS) were used. P is the proportion of true positive predictions to the total number of positive predictions. R is the proportion of true positive predictions in all positive samples. AP is the cumulative mean sum of PR curve areas under different confidence thresholds. To show the performance of different algorithms more comprehensively, two additional metrics were added [46]: all true (AT) and all miss (AM). AT is the proportion of images in which all the insulator defects are detected correctly. AM is the proportion of images in which all the insulator defects are not detected. For the mask extraction network, Dice coefficient was used to evaluate its performance, which is defined as,

(11) $$Dice\left(A,B\right)=\frac{2\left|A\cap B\right|}{\left|A\right|+\left|B\right|}$$

where A is the ground truth area and B is the area obtained by the mask extraction network.

## 5. Results and Discussions

To verify the effectiveness of our proposed methods, their performance was compared with five commonly used target detection methods, i.e., HOG + SVM [10], Haar + AdaBoost [11], Faster R-CNN [15], YoloV3 [24] and YoloV4 [47]. Meanwhile, to show the performance of our proposed methods more comprehensively, the results under two different backbones, i.e., ResNet50 and MobileNetV2 were also compared. The results when using images with different resolutions as inputs and when using different network structures are also presented in this section for a deeper understanding on our methods.

### 5.1. Determination of the Number of Residual Blocks

To determine the number of residual blocks between the encoding and decoding process of the U-Net in Figure 13, an analysis was conducted to examine the performances when using different number of residual blocks. The results shown in Table 3 indicate that the Dice coefficient gradually increases with the number of residual blocks in the rage of 0~3, but the Dice increase speed greatly slows down when the number of residual blocks continuously increase. As presented in Table 3, the Dice coefficients are similar when the number of blocks is higher than 3. Therefore, as introduced in Figure 13, three residual blocks were used.

### 5.2. Qualitative Detection Results

**Figure 19:**
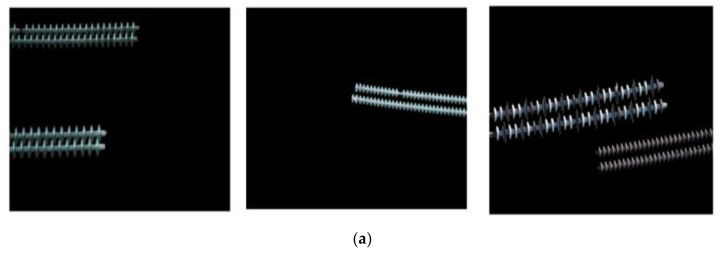

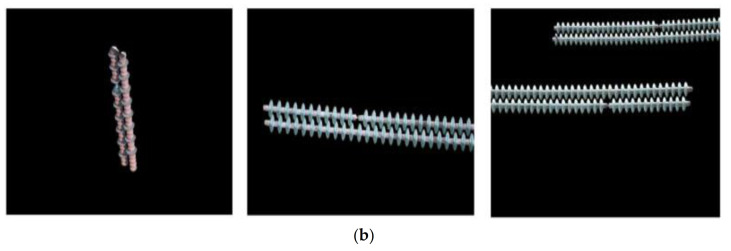
Mask examples mapped to the raw image after filtering under different backbones. (**a**) MobileNetV2. (**b**) ResNet.

Figure 19 shows the segmentation results of the mask extraction network mapped to the original image after denoising with morphological filter under the backbones of ResNet and MobileNetV2 respectively. We found that the improved mask extraction network based on U-Net has pretty good performance under different backbones. From our experimental data, the average Dice coefficient of the network is 0.97 on the backbone of ResNet and 0.94 on the backbone of MobileNetv2.

**Figure 20:**
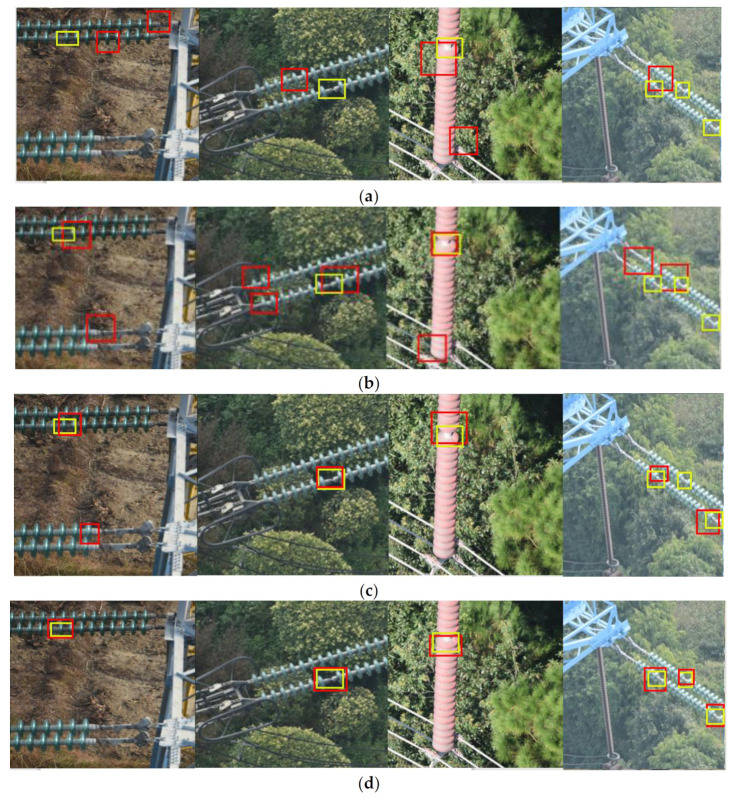

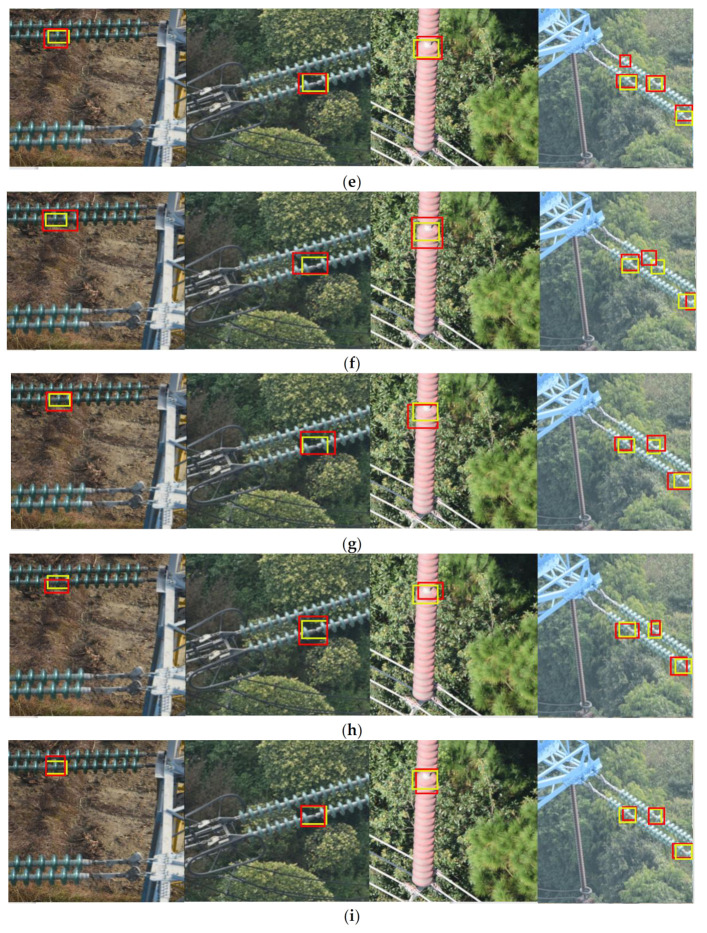
Qualitative results of all the examined methods. (**a**) HOG+SVM. (**b**) Haar+Adaboost. (**c**) YoloV3. (**d**) YoloV4. (**e**) Faster R-CNN. (**f**) ERCN-MobileNetV2. (**g**) ERCN-ResNet50. (**h**) CME-CNN-MoblieNetV2. (**i**) CME-CNN-ResNet50.

Figure 20 shows the qualitative results of all the methods examined on our dataset, where the yellow bounding box is the ground truth, and the red bounding box is the prediction. From Figure 18, a comparative summary can be concluded as follows.

Compared with the traditional vision methods, the method based on deep learning can detect more accurately and has less omissions and duplications. This is probably due to the fact that traditional vision-based methods usually use artificially designed features to represent objects and only consider some simple information (such as edge, color or texture) while the deep learning-based methods automatically extract both low-level and high-level features to represent the object. Therefore, the traditional vision method cannot effectively identify the insulator defects in situations with complex background.

Compared with Faster R-CNN and Yolov3, our proposed ERCN and CME-CNN have smaller prediction bias, higher positioning accuracy, and almost no omission and duplication. This is probably because our methods are based on multi-level classification and regression. Each cascade stage improves the detection quality by increasing the GIoU threshold, and introduce RoI Align to solve the mismatch problem, making the re-classification and re-regression network receive the correct features without position deviation. It makes the regression and classification effect better. In addition, in order to improve the detection performance of small targets, FPN is introduced which combines semantic features of different levels. These methods continue to strengthen the extraction and processing of image details, making the extracted target features completer and more accurate.

Compared with ERCN and Yolov4, there is almost no deviation between the prediction of CME-CNN and the ground truth. The detection performance of CME-CNN is better. This is probably because the complex background is simplified and the interference in detection is reduced by generating insulator mask image through the encoder-decoder mask extraction network. This makes the network more focused on the learning of target characteristics, making the network more efficient.

From the illustrated qualitative comparison results, we can conclude that the methods proposed in this paper are more accurate than the other methods mentioned above. There is basically no offset between the prediction and the ground truth, and the predictions are basically no omission or duplication. It can be seen that our method can better grasp the details of the image, can grasp more comprehensive and accurate features of the detected target.

### 5.3. Quantitative Detection Results

Table 4 gives the quantitative results of all the examined methods on our dataset. The results show that our proposed CME-CNN-ResNet50 has the best performance on five of the examined evaluation indexes (i.e., AP, P, R, AT and AM). Although with a not high FPS, it has great application value in practice because it does not require high real-time performance but requires high detection accuracy when using UAV to detect insulator defects. In addition, due to the introduction of depthwise separable convolution, ERCN-MobileNetV2 has the best FPS (i.e., 11.1). However, its detection performance is worse than CME-CNN-ResNet50. The FPS of CME-CNN-ResNet50 is 1.1, indicating that about one image can be processed in each second. This should be satisfactory for insulator defect detection using UAV because the detection using UAV does not have high requirements on the running time.

In general, the methods with higher detection accuracies usually need higher calculation costs [48]. For the insulator defect detection task in this study, the detection can be conducted on an expensive high-performance server in off-line practical applications. When an insulator defect is detected, it will be reported to the maintenance workers to manually replace the defect insulator. In this situation, the detection accuracy is the most important indicator, and the computation time is not as important as the detection accuracy. For example, the FPS of CME-CNN-ResNet50 is 1.1 and the FPS of YoloV4 is 10.6, which indicates that the computation time of CME-CNN-ResNet50 is 0.8 s slower than the number of YoloV4. In the above-mentioned application scenario, this 0.8 s doesn’t make any sense to speed up the maintenance that requires extensive human labor work. Therefore, in this study, we mainly focus on the detection accuracies of the examined methods.

### 5.4. Ablation Study Results

To examine how much the above-mentioned techniques contributed to the detection performance, an ablation study was conducted to compare the detection performance when using different skills. The results are given in Table 5, which shows that the use of cascade R-CNN increases the AP of the model by 1.9%, while the use of mask extraction increased the AP of the model by 4.2%. In addition, using both cascade R-CNN and mask extraction increases the R of the model by 5.8%.

**Figure 21:**
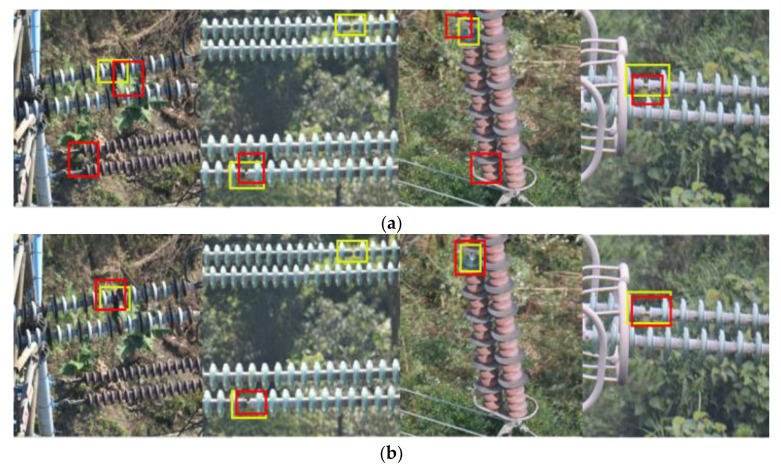

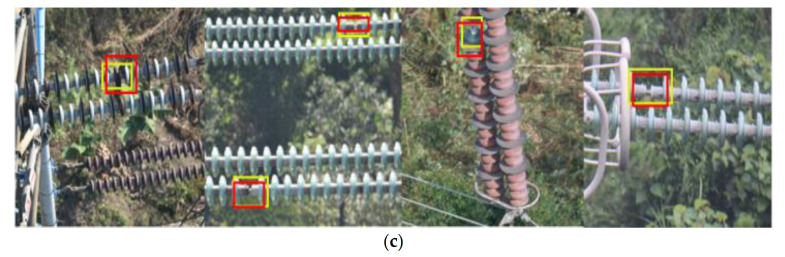
Ablation study results when using different techniques. (**a**) FPN+RA. (**b**) FPN+RA+CR. (**c**) FPN+RA+CR+ME. (FPN: Feature Pyramid Network. RA: RoI Align. CR: Cascade R-CNN. ME: Mask Extraction).

Figure 21 shows the influence of different techniques on the model performance. We can find that the use of cascade R-CNN can reduce the offset between the prediction and ground truth and makes the model prediction more accurate. The use of mask extraction makes the model more sensitive to the capture of insulator defects, better detection accuracy for small insulators, and the prediction basically has no offset with the ground truth.

### 5.5. Detection Performance on Images with Different Resolutions

The above experiments clearly show that the methods proposed in this paper can obtain high detection performance on high-resolution images. However, high-resolution images cannot always be available in practical applications because of many factors such as cost limitation, environmental condition, etc. [49]. Therefore, an experiment was conducted to compare the influence of image resolutions on the performance of CME-CNN-ResNet50 which performs the best on all the examined indexes except FPS in Table 4. 

**Figure 22:**
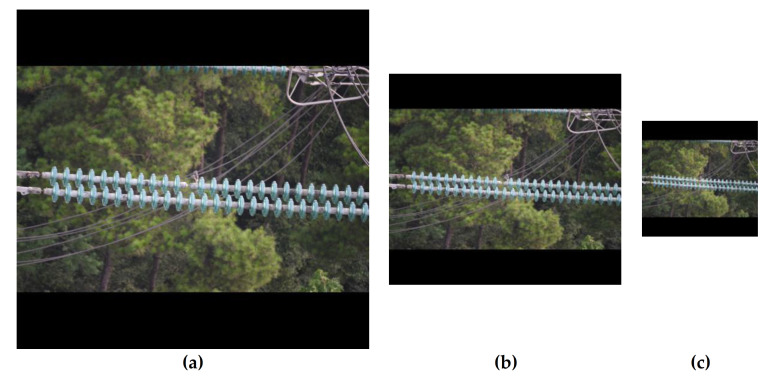
Examples of high, medium and low resolution images. (**a**) High. (**b**) Medium. (**c**) Low.

The compression method mentioned in Section 4 was used to compress the image of our dataset to 512 × 512 and 256 × 256. Examples of the compressed images are shown in Figure 22. The compressed 512 × 512 images are regarded as medium-resolution images and the compressed 256 × 256 images are regarded as low-resolution images. The comparison results are shown in Table 6. The results show that the lower the resolution of the test images, the higher the FPS of the network. However, the lower resolution of the test images leads to lower values of the examined AP, P, R, AT, and AM. The AP on high-resolution images is 5.1% and 2.2% higher than the numbers on medium- and low-resolution images, respectively. This is mainly due to the information loss on small targets such as insulator defects in lower resolution images.

### 5.6. Detection Performance When Using Different ResNet Structures

Besides image resolution, the ResNet structure also has an impact on the detection performance. In [50], ResNet-v2 was proposed. Different from ResNet-v1, ResNet-v2 puts the activation function on the residual branch, which makes the computational speed faster in the back propagation and forward propagation. Here in this study, another experiment was conducted to compare the performance of different ResNet structures on the error rate of the model. Table 7 shows the error rates when using ResNet50-v1 or ResNet50-v2. The results show that the error rates of Faster R-CNN, ERCN, CME-CNN in ResNet50-v2 are reduced by 1.7%, 1.9%, 2.1%, respectively. This indicates that our proposed method can be further improved by alternatively using ResNet50-v2. 

### 5.7. Novelty and Application of our Proposed Methods

Different from the traditional one-stage or two-stage detection methods, our proposed methods use multi-level classification and regression as well as information fusion and detect based on the mask image. On the one hand, multi-level classification and regression improves the quality of prediction box by increasing the GIoU threshold. Our methods also use RoI Align for allowing the selected RoI receive a correct feature without position bias, making a better regression and classification results. On the other hand, information fusion uses FPN network to combine different levels of semantic features to enhance the extraction of bottom details. When stitching the underlying and advanced features in U-Net, the SE block is used to assign different weights to each channel, and different attention is given to low-level features and high-level features. Residual block is also adopted to make the network learn deeper features. More importantly, the interference of complex background is eliminated after obtaining the mask image, which makes the detection more targeted and makes the network focus on training target features. Due to the introduction of depthwise separable convolution, our proposed methods even can be used for real-time detection. 

Therefore, our model method CME-CNN-ResNet50 can be used for high-precision inspection tasks such as insulator defects. In addition, in the case of different backbones such as MobileNetv2 and ResNet50, our model can have a good trade-off between detection accuracy and running speed and can be applied to detection scenarios with different demands.

## 6. Conclusions

In this paper, two methods (i.e., ERCN and CME-CNN) for insulator defect detection based on Faster R-CNN are proposed to be applied to high-resolution aerial images. Our proposed methods in two different backbones (i.e., ResNet50 and MobileNetV2) are used to compare with five different detection methods (i.e., HOG+SVM, Haar+AdaBoost, Faster R-CNN, YoloV3, and YoloV4). The results show that the average precision (AP) of our proposed CME-CNN-ResNet50 achieves the best performance (88.7%) which is 52.9%, 58.2%, 6.5%, 10.8%, and 1.6% higher than the number of HOG+SVM, Haar+AdaBoost, Faster R-CNN-ResNet50, YoloV3, and YoloV4, respectively. The ablation study results show that the mask extraction proposed in CME-CNN contributes to a 4.2% increase on AP than without using this skill and using both cascade R-CNN and mask extraction increases the AP by 7.1%. Besides, the results also show that the performance of our proposed method can be further improved by alternatively using ResNet50-v2. However, the proposed methods are based on insulator images captured in daytime, while detecting insulator defects at night is still a challenging task. Therefore, one of our future work will focus on improving the night detection performance by using image enhancement technologies [51]. Moreover, researchers have introduced the concept of Corner Net [52] and Extreme Net [53] into target detection in the recent years, and satisfactory results have been reported. In our future work, we will also try to combine our proposed method with the detection of key points to see whether we can further improve the detection performance of our network.

## Figures and Tables

**Table 1:** A brief summary of the previous related methods.

Method	Merits	Demerits	Studies
physical methods	not affected by complex visual background	laborious; easy to be affected by meteorological conditions, surrounding heat source, and other factors.	[3,4]
traditional vision-based methods	high efficiency, low computational burden	easy to be affected by the complex background; depending on accumulated experience to extract image features	[5,6,7,8,9,10,11]
deep learning-based methods	high accuracy and feature extraction ability	depending on large amounts of data.	[12,13,14,15,16,17,18,19,20]

**Table 2:** Comparison Between Different Methods.

Model	BB	BN	FPN	CR	IRS	Conv method
R-CNN	AlexNet	×	×	×	×	Std-Conv
Fast R-CNN	Vgg16	×	×	×	×	Std-Conv
Faster R-CNN	Vgg16	√	×	×	×	Std-Conv
ERCN	ResNet50	√	√	√	×	Std-Conv
CME-CNN	MobileNetV2	√	√	√	√	Dwise-Conv

BB: Backbone. BN: Batch Normalization. IRS: Inverted Residuals. FPN: Feature Pyramid Network. CR: Cascade R-CNN. Dwise-Conv: depthwise separable convolution.

**Table 3:** The relationship between the number of residual blocks and Dice coefficient.

**Number of Residual Blocks**	0	1	2	3	4	5	6
**Dice Coefficient**	89.4	90.8	92.7	94.9	95.2	95.4	95.5

**Table 4:** Quantitative results of all the examined methods.

Method	AP	P	R	AT	AM	FPS
HOG+SVM	35.8	56.5	45.1	33.2	13.4	-
Haar+Adaboost	30.5	49.2	42.2	37.5	30.5	-
YoloV3	77.9	81.1	79.7	78.8	7.7	10.2
YoloV4	87.1	89.8	88.9	88.1	0.67	10.6
Faster R-CNN-ResNet50	82.2	84.4	82.9	81.6	2.2	3.7
ERCN-ResNet50	84.5	87.7	86.8	86.2	1.7	3.2
CME-CNN-ResNet50	**88.7**	**91.2**	**90.1**	**88.6**	**0.62**	1.1
ERCN-MobileNetV2	83.1	86.9	85.4	85.2	1.9	**11.1**
CME-CNN-MoblieNetV2	86.9	89.3	88.6	87.8	0.75	3.6

The bold number indicates the best method for each of the examined indexes.

**Table 5:** Detection results using different techniques in our network.

FPN	RA	CR	ME	P	R	AP
√	√	×	×	85.9	84.3	81.6
√	√	√	×	87.7	86.8	84.5
√	√	√	√	**91.2**	**90.1**	**88.7**

FPN: Feature Pyramid Network; RA: RoI Align; CR: Cascade R-CNN; ME: Mask Extraction. The bold number indicates the best method for each of the examined indexes.

**Table 6:** Detection results when using images with different resolutions.

Resolution	AP	P	R	AT	AM	FPS
High	**88.7**	**91.2**	**90.1**	**88.6**	**0.62**	1.1
Medium	86.5	88.2	87.1	85.9	0.75	2.5
Low	83.6	86.2	84.4	82.3	1.15	**3.6**

**Table 7:** Detection error rates when using different ResNet structures.

Structure	Faster R-CNN	ERCN	CME-CNN
ResNet50-v1	15.6	12.3	8.8
ResNet50-v2	13.9	10.4	6.7

## Data Availability

The data used in this study is publicly available online [33].

## Abbreviations

The following abbreviations are used in this manuscript:

UAV	Unmanned Aerial Vehicle
R-CNN	Region-based Convolutional Neural Network
FPN	Feature pyramid network
GIoU	Generalized Intersection over Union
RoI	Region of Interest
IoU	Intersection over Union
ERCN	Exact R-CNN
RPN	Regional recommendation network
FCN	Fully Convolutional Network
R-FCN	Region-based Fully Convolutional Network
Yolo	You only look once
SSD	Single Shot multibox Detector
HOG	Histogram of Oriented Gridients
SVM	Support Vector Machine
DCNN	Dynamic Convolutional Neural Network
RA	RoI Align
CR	Cascade R-CNN
ME	Mask Extraction
CME-CNN	Cascade the Mask Extraction and exact region-based Convolutional Neural Network
